# Concomitant Systolic and Diastolic Doppler Alternans: An Ominous Sign of Left Ventricular Dysfunction

**DOI:** 10.7759/cureus.71074

**Published:** 2024-10-08

**Authors:** Luai Madanat, Ahmad Jabri, Richard Bloomingdale, Abhay Bilolikar

**Affiliations:** 1 Internal Medicine, Beaumont Hospital, Royal Oak, USA; 2 Cardiology, Beaumont Hospital, Royal Oak, USA

**Keywords:** doppler alternans, echocardiogram, heart failure, pulsus alternans, severe left ventricular dysfunction

## Abstract

Pulsus alternans, defined by an arterial pulse alternating between strong and weak beats, often signify severe left ventricular dysfunction. Detectable on physical examination or by echocardiography, this phenomenon highlights substantial cardiac compromise. We discuss a case involving a 59-year-old male with non-ischemic cardiomyopathy who was admitted for acute congestive heart failure. The echocardiogram revealed severe left ventricular dysfunction with systolic and diastolic beat-to-beat variations in blood flow on Doppler, a phenomenon known as Doppler alternans. Notably, these echocardiographic findings preceded any clinical signs, leading to a timely escalation in care. This case emphasizes the critical role of echocardiography in assessing ventricular function and providing prognostic insights that can influence management strategies.

## Introduction

Pulsus alternans is characterized by an arterial pulse with alternating strong and weak beats. This hemodynamic phenomenon is commonly associated with severe ventricular dysfunction signaling a poor prognosis and the potential for pulseless electrical activity [1]. This variation in pulse amplitude can be observed through physical exams or via echocardiogram, a phenomenon known as Doppler alternans [2]. The underlying mechanisms of pulsus alternans have been explored through two main theories. The first suggests that alternans result from changes in cardiac filling volumes; greater diastolic volumes lead to stronger contractions, while reduced systolic and diastolic volumes result in weaker subsequent beats. The second theory, which is load-independent, involves disturbances in cellular calcium handling, a phenomenon observed in the cardiomyocyte studies of affected patients [3,4].

Echocardiography plays a crucial role in heart failure diagnosis, management, and prognostication, offering more than just ejection fraction measurements. Its versatility allows for the comprehensive assessment of cardiac volumes, diastolic function, right ventricular performance, hemodynamics, and valvular regurgitation, making it an invaluable tool in evaluating multiple aspects of heart function. This case illustrates severe left ventricular dysfunction with Doppler alternans evident on echocardiography before clinical signs, underscoring the critical role of echocardiography in assessing ventricular function beyond just ejection fraction measurement.

## Case presentation

**Figure 1:**
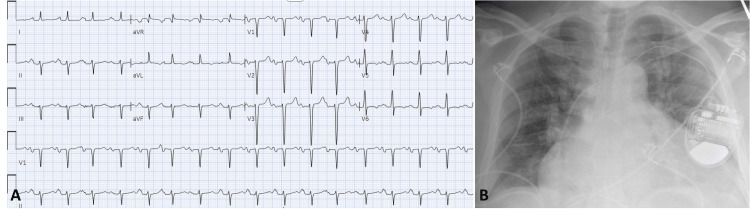
Initial workup. A: Electrocardiogram demonstrating sinus tachycardia with premature atrial contractions, bi-atrial enlargement present. B: Chest x-ray showing cardiomegaly and pulmonary edema.

A 59-year-old male with a history of non-ischemic cardiomyopathy and a left ventricular ejection fraction (LVEF) of 20% status post primary prevention single-chamber implantable cardioverter-defibrillator (ICD), three years prior, presented with a three-day history of worsening dyspnea. On examination, he was tachycardic with heart rates around 120 bpm with normal blood pressure and exhibited signs of volume overload. ECG showed sinus tachycardia with premature atrial contractions and bi-atrial enlargement (Figure 1A). Chest X-ray revealed cardiomegaly and pulmonary edema (Figure 1B). Lab results were notable for elevated creatinine at 2.2 mg/dL and a BNP (B-type natriuretic peptide) level of 2068 pg/mL. He was admitted with acute congestive heart failure and started on aggressive diuresis and medical management.

**Figure 2:**
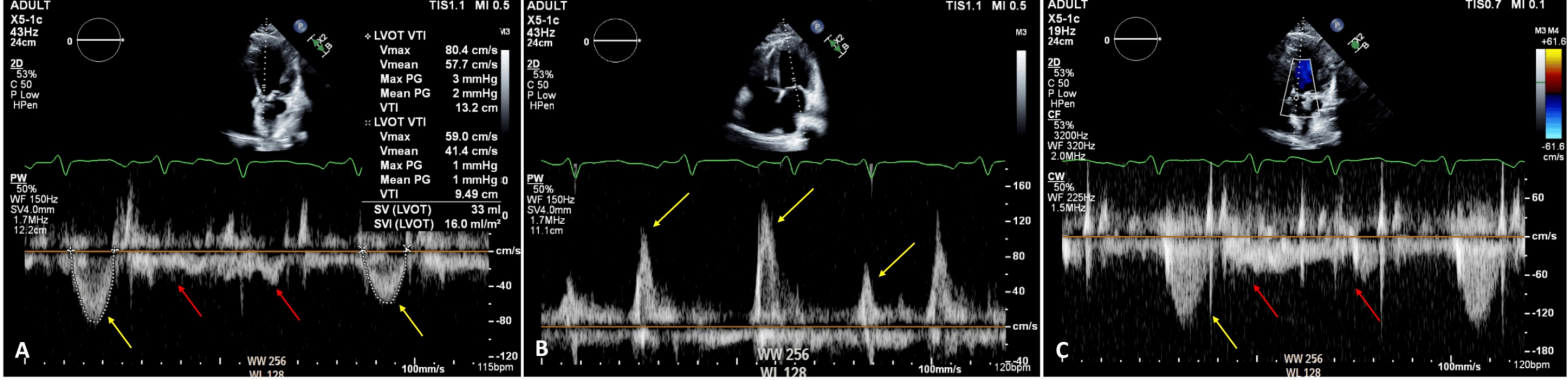
Echocardiographic findings. A: Left ventricular (LV) outflow tract pulsed wave Doppler showing beat-to-beat variation in systolic flow (yellow arrow). Notice significantly reduced absent flow in multiple consecutive beats (red arrow). B: Pulsed wave Doppler through mitral valve demonstrating diastolic beat-to-beat variation in LV inflow (yellow arrow). C: Continuous wave Doppler through the aortic valve demonstrating Doppler alternans (yellow arrow) and significantly reduced systolic flow on multiple beats (red arrow).

Transthoracic echocardiography (TTE) revealed a severely reduced left ventricular ejection fraction (LVEF) of 15%, with moderate mitral and tricuspid valve regurgitation. Pulsed-wave (PW) doppler of the left ventricular outflow tract (LVOT) demonstrated beat-to-beat variation in systolic blood flow, which was also observed in diastolic flow through the mitral valve (Figure 2A, 2B). Continuous-wave (CW) Doppler across the aortic valve confirmed severely reduced or absent flow on multiple beats (Figure 2C). Due to these findings and his overall clinical status, the patient was transferred to the cardiac intensive care unit.

**Figure 3:**
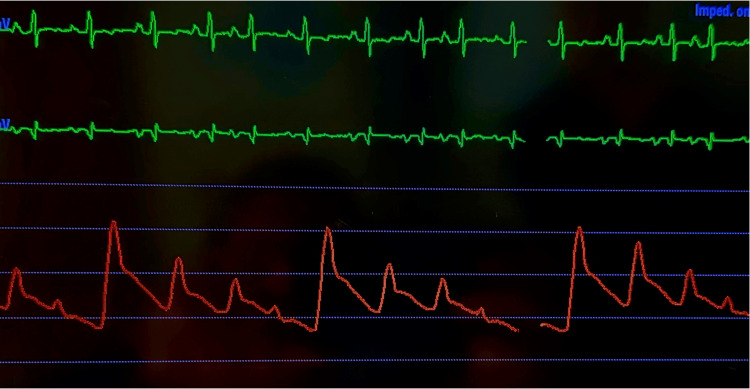
Invasive hemodynamics. Arterial line tracing demonstrating significant pulses alternans.

Arterial line monitoring demonstrated low blood pressure with pulsus alternans, confirming the concern for severe left ventricular dysfunction (Figure 3). Milrinone infusion was initiated, leading to a satisfactory clinical response. A right heart catheterization revealed a pulmonary capillary wedge pressure of 20 mmHg, a right atrial pressure of 12 mmHg, a cardiac output of 2.9 L/min, and a cardiac index of 1.4 L/min/m². The patient eventually underwent biventricular assist device implantation during his hospital stay.

## Discussion

Pulsus alternans, characterized by alternating strong and weak arterial pulse beats, typically signals severe left ventricular (LV) dysfunction [3]. It is usually seen in patient with severe LV systolic rather than diastolic heart failure. This phenomenon can be indirectly observed through doppler analysis, known as doppler alternans [2]. The most plausible explanation involves abnormal intracellular calcium handling in the failing myocardium, although the precise mechanisms remain unclear [5,6]. The presence of pulsus alternans indicates terminal systolic heart failure and is associated with a poor prognosis [4,6]. Previous studies have shown that inotropic agents can improve pulsus alternans in heart failure patients, suggesting that addressing acute decompensation can enhance cardiac performance and potentially reverse this condition [2]. Furthermore, pulsus alternans have been documented in acute heart failure cases due to pulmonary embolism, aortic stenosis, and coronary ischemia [7-9]. Irrespective of the underlying etiology, the detection of pulsus alternans should raise clinical concern and encourage prompt management.

Although our patient was known to have end-stage heart failure (stage D) due to dilated cardiomyopathy, prior echocardiograms during outpatient visits did not demonstrate any evidence of Doppler alternans despite a known baseline ejection fraction of 20%. However, during this admission, in the setting of acute decompensation and worsening heart failure, echocardiographic Doppler revealed both systolic and diastolic beat-to-beat flow variation, indicative of severe LV failure, which resolved with appropriate treatment. This is in line with prior studies that have demonstrated the reversibility of these findings with appropriate heart failure treatment [2]. In addition, pulsus alternans were not detected on physical exam but the presence the presence of Doppler alternans raised concerns, leading to early intervention before further clinical decline. This case highlights the value of echocardiographic findings, such as Doppler alternans, in providing prognostic insights beyond LVEF, enabling timely interventions.

## Conclusions

Pulsus alternans is a clinical sign commonly associated with severe systolic heart failure, particularly in cases of advanced left ventricular (LV) dysfunction. It is characterized by alternating strong and weak arterial pulses with each heartbeat, reflecting the heart's inability to maintain consistent contractile strength. This phenomenon is often a marker of worsening systolic function and indicates a poor prognosis. Pulsus alternans are thought to result from impaired calcium cycling within the cardiac myocytes, leading to variability in contraction strength between beats. While it can be detected on physical examination, it can be first identified through echocardiography or Doppler analysis, where it is termed Doppler alternans. The presence of pulsus alternans in severe heart failure patients highlights the need for prompt medical intervention, as it signifies advanced myocardial failure and the potential for rapid clinical deterioration. Timely recognition and treatment can improve hemodynamic stability, especially through the use of inotropic agents or other heart failure therapies.
